# Ghrelin-O-Acyltransferase (GOAT) Enzyme as a Novel Potential Biomarker in Gastroenteropancreatic Neuroendocrine Tumors

**DOI:** 10.1038/s41424-018-0058-8

**Published:** 2018-10-08

**Authors:** Aura D. Herrera-Martínez, Manuel D. Gahete, Rafael Sánchez-Sánchez, Emilia Alors-Perez, Sergio Pedraza-Arevalo, Raquel Serrano-Blanch, Antonio J. Martínez-Fuentes, Maria A. Gálvez-Moreno, Justo P. Castaño, Raúl M. Luque

**Affiliations:** 1 Maimonides Institute for Biomedical Research of Cordoba (IMIBIC), Córdoba, Spain; 2 Endocrinology and Nutrition Service, Reina Sofia University Hospital, Córdoba, Spain; 3 Department of Cell Biology, Physiology, and Immunology, University of Córdoba, Córdoba, Spain; 4 CIBER Fisiopatología de la Obesidad y Nutrición (CIBERobn), Córdoba, Spain; 5 Reina Sofia University Hospital, Córdoba, Spain; 6 Pathology Service, Reina Sofia University Hospital, Córdoba, Spain; 7 Medical Oncology Service, Reina Sofia University Hospital, Córdoba, Spain

## Abstract

**Objectives:**

The association between the presence and alterations of the components of the ghrelin system and the development and progression of neuroendocrine tumors (NETs) is still controversial and remains unclear.

**Methods:**

Here, we systematically evaluated the expression levels (by quantitative-PCR) of key ghrelin system components of in gastroenteropancreatic (GEP)-NETs, as compared to non-tumor adjacent (NTA; *n* = 42) and normal tissues (NT; *n* = 14). Then, we analyzed their putative associations with clinical-histological characteristics.

**Results:**

The results indicate that ghrelin and its receptor GHSR1a are present in a high proportion of normal tissues, while the enzyme ghrelin-O-acyltransferase (GOAT) and the splicing variants In1-ghrelin and GHSR1b were present in a lower proportion of normal tissues. In contrast, all ghrelin system components were present in a high proportion of tumor and NTA tissues. GOAT was significantly overexpressed (by quantitative-PCR (qPCR)) in tumor samples compared to NTA, while a trend was found for ghrelin, In1-ghrelin and GHSR1a. In addition, expression of these components displayed significant correlations with key clinical parameters. The marked overexpression of GOAT in tumor samples compared to NTA regions was confirmed by IHC, revealing that this enzyme is particularly overexpressed in gastrointestinal NETs, where it is directly correlated with tumor diameter.

**Conclusions:**

These results provide novel information on the presence and potential pathophysiological implications of the ghrelin system components in GEP-NETs, wherein GOAT might represent a novel diagnostic biomarker.

## Introduction

Neuroendocrine tumors (NETs) comprise a heterogeneous family of malignancies with complex clinical behavior and increasing incidence^1–3^. Primary tumor is identified only in 70% of patients^4^, while distant metastases are frequently found at diagnosis (27–73%), influencing the overall survival^5–7^. Despite that histological differentiation and Ki67 index are some prognosis factors^4^, well-differentiated low-grade tumors may behave aggressively^8^. Unfortunately, surgery is often not applicable since most tumors are diagnosed at advanced stage. For these reasons, the development of novel diagnostic markers has gained scientific and clinical interest^9,10^.

The ghrelin system is involved in the regulation of multiple (patho)-physiological functions, including hormonal secretion, β-cell survival or appetite and gastric motility^11–14^. Ghrelin must undergo a unique modification, consisting of the acylation of the third serine residue, which is catalyzed by the ghrelin-O-acyltransferase (GOAT) enzyme^14,15^. Acylated ghrelin (AG) represents the peptide binding and activating its canonical ghrelin receptor, GHSR1a. Interestingly, several ghrelin system variants, resulting from post-transcriptional modifications or alternative splicing, have been identified, including the In1-ghrelin^11,16^ and a truncated receptor GHSR1b, with unknown ligand and function^11,16,17^.

Alterations in the expression of specific components of this system have been associated with the development/progression of various neoplasms^16,18–21^, including NETs, but the clinical-molecular correlations have not been elucidated^22,23^. Accordingly, in this study we aimed to: (1) analyze systematically the expression of different components of ghrelin system in gastroenteropancreatic-(GEP-)NETs compared to non-tumor adjacent (NTA) tissue and, most importantly, to normal control tissues by quantitative real-time PCR (qPCR); (2) correlate the expression of these components with clinical/histological characteristics; and (3) perform in vitro experiments to elucidate the potential pathophysiological role of GOAT enzyme as a key component particularly altered in our cohort of NET samples, using BON-1 and QGP-1 cell lines.

## Materials and methods

### Patients and samples

This study was approved by the Ethics Committee of the Reina Sofia University Hospital (Cordoba, Spain), was performed according to the Declaration of Helsinki, and patients were treated following national and international clinical practice guidelines. A written informed consent was required before inclusion. Data from 42 patients with GEP-NETs were collected (demographic and clinical characteristics of the cohort are summarized in Table 1). Additionally, 14 normal control tissues from healthy donors were also included. Patients with hereditary endocrine syndrome were excluded. Clinical records were used to collect full medical history. GEP-NETs were classified according to histopathology features as well-differentiated NETs (G1), moderately differentiated (G2), and poorly differentiated NETs (G3)^24^. Formalin-fixed paraffin-embedded (FFPE) samples were also collected (42 tumor samples, 42 NTA and 14 normal tissues).

**Table 1:** General characteristics of the patient population

General characteristic		% (*n*)
Sex		
Male		52.4% (22)
Female		47.6% (20)
Age at diagnosis		55.66 ± 17 years
Personal history of other tumors		15.0% (6)
Smoke habit		
Active		45.0% (9)
Ex-smoker		20.0% (4)
No habit		35.0% (7)
Family history of neoplasms		52.9% (9)
Incidental tumor		37.9% (11)
Functionality		43.3% (13)
Mortality rate		18.9% (7)

### RNA isolation and reverse-transcription

Total RNA from FFPE samples (*n* = 98) was isolated using the RNeasy-FFPE Kit (Qiagen, Limburg, Netherlands) according to the manufacturer’s instructions. Quantification of the recovered RNA was assessed using NanoDrop2000 spectrophotometer (Thermo Scientific, Wilmington, NC). Total RNA was retrotranscribed to cDNA with the First-Strand Synthesis kit using random hexamer primers (Thermo Scientific) as previously reported^25–28^

### Quantitative real-time PCR (qPCR)

cDNAs were amplified with the Brilliant III SYBR-Green Master Mix (Thermo Scientific) using the Stratagene Mx3000p system and specific primers for each transcript of interest. Specifically, expression levels (absolute mRNA copy number/50 ng of sample) of ghrelin, In1-ghrelin, GOAT enzyme, GHSR1a and GHSR1b, were measured using previously validated primers^21,29,30^. RNA expression was adjusted by *18S* gene expression^28,31^.

### Immunohistochemistry (IHC) analysis

IHC analysis of GOAT was implemented in all 42 FFPE samples (tumor and NTA regions) using standard procedures^32^. Optimum antibody concentration (1:300) using a commercially available antibody against human GOAT (AA257-287, Acris-antibodies, Herford, Germany) was selected by performing a series of antibody dilution tests in normal pancreas^33^. Two independent pathologists performed the IHC analysis following a blinded protocol. In the analysis, 0, 1 + , 2 + , 3 + stand for absent, low, moderate, and high staining intensities of GOAT enzyme in the tumor compared to the NTA region.

### Cell culture

In vitro experiments were performed using human NET cell lines BON-1^34^ and QGP-1^35^. BON-1 cells were cultured in DMEMF12 (Life Technologies, Barcelona, Spain) supplemented with 10% fetal bovine serum (FBS; Sigma-Aldrich, Madrid, Spain), 1% glutamine (Sigma-Aldrich) and 0.2% antibiotic (Gentamicin/Amphotericin-B; Life Technologies). QGP-1 cells were cultured in RPMI-1640 (Lonza, Basel, Switzerland), supplemented with 10% FBS, 1% glutamine, and 0.2% antibiotic. Both cells lines were cultured at 37  °C in a 5% CO_2_ incubator and monthly checked for mycoplasma contamination by PCR^36^.

### Cell proliferation assay in response to GOAT inhibitor

The only commercially available GOAT inhibitor (GOATi; GO-CoA-Tat; Ref: 032–37) was purchased from Phoenix Pharmaceuticals (Burlingame, CA). The final concentration (10^-5 ^M) was selected based on dose–response experiments performed in prostate cell-lines and on previous reports^37^. Cell proliferation was determined by using Alamar-blue assay (basal, 24, 48, and 72 h) as previously reported^21,22,32^. Cells were seeded per quadruplicate and assays were repeated four times. Paclitaxel (PAX; Sigma-Aldrich) was used as control for the inhibition of proliferation^27,30^.

### Migration capacity assay

The ability of BON-1 cells to migrate after 24 h of treatment was evaluated by wound-healing technique^22,38–40^. Briefly, stable cells were plated at sub-confluence in 6-well plates. The wound was made on confluent cells using a 100 μl sterile pipette tip. Wells were rinsed in PBS and treated for 24 h in FBS-free medium. Wound-healing was calculated as the area of a rectangle centered in the picture 24 h after the wound vs. the area of the rectangle just after doing the wound. Three experiments were performed in independent days, in which three random pictures per well along the wound were acquired and, the mean area of these pictures was used for analysis. Images were analyzed using the ImageJ software^41^.

### Statistical analysis

Paired *t*-test analysis was used to compare the expression levels between GEP-NETs samples and NTA tissue. Non-paired *t*-test analysis was used to compare the expression levels between normal tissue and GEP-NETs samples or NTA tissue. *U*-Mann–Whitney tests were used to evaluate clinical-molecular relations within GEP-NETs samples. Chi-squared test was used to compare categorical data. All statistical analyses were performed using SPSS and GraphPad Prism. Data are expressed as mean ± SEM. *p*-values < 0.05 were considered statistically significant. In functional experiments, results were expressed as percentage vs. control (vehicle-treated cells). Cell proliferation rate compared to control was assessed by multiple comparison test (two-way ANOVA followed by Newman-Keuls post-hoc test).

## Results

Forty-two patients with GEP-NETs were included. Demographic/clinical features are summarized in Table 1. Specifically, 15 patients presented PNETs and 27 patients presented gastrointestinal (GI)-NETs [52.3% males (22/42); mean age 55.6 ± 17 years]. Tumor characteristics are summarized in Table 2. In our cohort, 43.3% (13/30) were functioning tumors; 63.2% (24/38) had peritumoral invasion [34.3% (12/35) vascular and 35.3% (12/34) neural invasion], 52.4% (22/42) had metastasis at diagnosis [multiple localization in 36.3% (8/22)], 63.2% (24/38) were invasive tumors and the mortality rate reached 18.9% (7/37). Relapsed disease was observed in 36% of patients (13/36). Finally, almost 70% of samples (29/42) were considered as low/intermediate tumors [38.1% (16/42) grade 1 and 31% (13/42) grade 2]. PNETs were statistically larger in size compared to those GI-NETs (4.0 ± 0.47 vs. 2.36 ± 0.34 cm, respectively; *p* < 0.01).

**Table 2:** Tumor sample characteristics

Characteristic		% (*n*)
Tissue samples		
Primary tumor		42
Non-tumor adjacent tissue		42
Normal tissue		14
Primary tumor localization		
Pancreas		35.7% (15)
Stomach		7.1% (3)
Small bowel		31.0% (13)
Colon and rectum		26.2% (11)
Maximal tumor diameter		2.98 ± 1.86 cm
Pancreas NETs		4.0±0.47 cm
Gastrointestinal NETs		2.36±0.34 cm
Necrosis		
<10%		16.7% (1)
10–20%		16.7% (1)
21–30%		50% (3)
> 30%		16.7% (1)
Depth of infiltration (gastrointestinal NETs)		
Submucosa		4.5% (1)
Mucosa		4.5% (1)
Muscular		40.9%(9)
Serosa		50.0%(11)
Multiple tumors		8.0% (2)
Peritumoral tissue invasion		63.2% (24)
Vascular invasion		34.3% (12)
Neural invasion		35.3% (12)
Metastasis		52.4% (22)
Metastasis localization		
Liver		9.1% (2)
Lymphatic nodules		54.5% (12)
Multiple		36.3% (8)
Grading (WHO 2010 criteria)		
Low		38.1% (16)
Intermediate		31.0% (13)
High		4.8% (2)
Unknown		26.2% (11)
Post-surgical treatment		43.2% (16)
Relapsed disease		36.1% (13)
Disease free		55.9% (19)
New surgery requirements		18.2% (6)

### Histopathological characterization of GEP-NETs and NTA tissue

Primary tumor samples were delimited from the NTA tissues after the evaluation of two experienced pathologists using histology and immunohistochemistry, as previously reported^32^.

### Expression of components of the ghrelin system in control and GEP-NETs samples

**Fig. 1:**
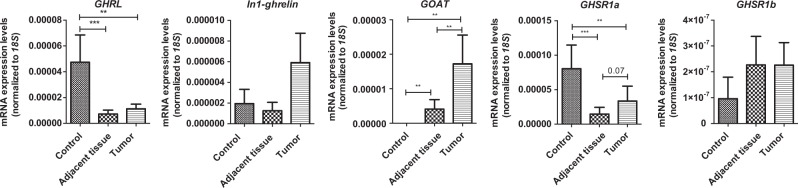
Expression of ghrelin system components in normal GEP, adjacent non-tumor tissue and GEP-NETs. The absolute mRNA expression of the different components of the ghrelin system was determined by qPCR in normal GEP controls, adjacent non-tumor tissue and GEP-NETs samples (values are adjusted by *18S* expression). Data represent the mean ± SEM. Asterisks (*p < 0.05; **p < 0.01; ***p < 0.001) indicate significant changes by paired analysis between adjacent non-tumor and GEP-NETs and non-paired analysis between normal tissue and adjacent non-tumor or tumor tissues

Ghrelin system components were present at variable proportions in normal GEP samples, as determined by qPCR. Ghrelin and its native receptor GHSR1a were expressed in more than 80% of healthy controls (34/42 and 39/42, respectively), while their splicing variants In1-ghrelin and GHSR1b were expressed in about 40% of the samples (17/42 and 19/42, respectively). In contrast, expression of GOAT enzyme was only detected in less than 20% (7/42) of normal samples (Supp. Fig. 1). Ghrelin and GHSR1a were also present in a high proportion (more than 60%) of the NTA and tumor samples (32/42 and 29/42, respectively); while GOAT enzyme and the splicing variants In1-ghrelin and GHSR1b were present in more than 40% of the samples (25/42, 21/42 and 17/42, respectively; Supp. Fig. 1). Of note, ghrelin expression levels were decreased in NTA and tumor tissue compared with normal samples, with a slightly but not significantly increased expression in tumor compared with NTA tissue (Fig. 1). A similar observation was found for GHSR1a expression, while GOAT enzyme was clearly overexpressed in tumor tissues compared with NTA regions and normal tissues, wherein it was virtually absent (Fig. 1). Finally, In1-ghrelin was more expressed in tumor tissues than in control samples but these differences were not statistically significant, while no significant changes were found in the case of the splicing variant GHSR1b (Fig. 1).

In terms of tumor grade, no significant differences in the expression of any of the ghrelin system components analyzed were found between differentiated (G1/G2) and non-differentiated (G3) GEP-NET (Supp. Fig. 2). However, we found that the expression of GOAT enzyme and GHSR1a in GI-NETs was markedly higher than in PNETs, while the expression of ghrelin was lower in GI-NETs compared to PNETs (Supp. Fig. 3). Additionally, ghrelin expression levels correlated with those of In1-ghrelin (*R*^2^ = 0.532; *p* < 0.01) and GOAT (*R*^2^ = 0.422; *p* < 0.05) in tumor samples, while the expression of GHSR1a was correlated with GHSR1b (*R*^2^ = 0.444; *p* < 0.05).

### Immunohistochemistry analysis of the presence of GOAT enzyme

**Fig. 2:**
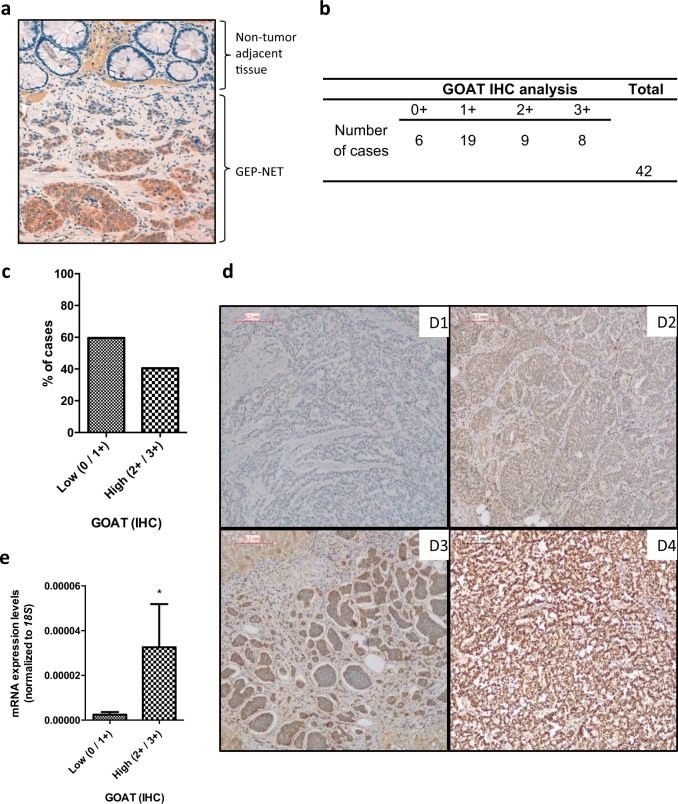
Immunohistochemical analysis of GOAT enzyme in adjacent non-tumor tissue and GEP-NETs. The presence of GOAT by immunohistochemistry using a specific antibody was determined in a subset of samples, which included tumor and non-tumor regions from patients diagnosed with GEP-NETs. **a** Representative images of the IHC analysis of GOAT enzyme in a GEP-NET sample compared with the non-tumor adjacent tissue. **b** Absolute number of cases according to the intensity of GOAT IHC staining (0, 1 + , 2 + , 3 + ). **c** The graph indicates the percentage of tumor samples according to the intensity of GOAT expression by IHC, 0 and 1 + have been grouped as low expression while 2 + and 3 + have been grouped as high intensity by IHC. **d** Representative images of different GOAT staining in GEP-NETs. In the analysis, 0, 1 + , 2 + , 3 + stand for absent, low, moderate, and high intensities of the tumor region staining compared to the adjacent region with non-tumor tissue (3D1, 3D2, 3D3, 3D4, respectively). This analysis revealed that GOAT was present in the vast majority of tumor cells compared with non-tumor adjacent tissue, with different grades of staining. **e** Correlation between the absolute mRNA expression of GOAT determined by qPCR in GEP-NETs samples (values are adjusted by *18S* expression) and the intensity of GOAT staining

Based on the marked overexpression of GOAT enzyme, as well as on previous reports^16,18^, we also sought to analyze its presence at the protein level. IHC analysis of tumor tissue revealed that GOAT enzyme was present in the vast majority of tumor cells compared with NTA tissue (Fig. 2a), with different grades of staining. Indeed, in our cohort, 86% of the tumor samples (36/42) evaluated were positive for the presence of GOAT enzyme by IHC (Fig. 2b), wherein 40% of the tumor cases (17/42) presented a strong staining (2 + or 3 + ) for GOAT compared to NTA tissue (Fig. 2c, d). Of note, mRNA expression levels in tumors samples correlated with GOAT expression by IHC (Fig. 2e). Additionally, strong staining (2 + or 3 + ) for GOAT was correlated to increased age at diagnosis (62.5 ± 4 years) compared to those tumors with absent or lower staining (0 or 1 + ; 51 ± 2 years; *p* > 0.05).

### Correlations between the expression levels of ghrelin system components and clinical-histological characteristics in GEP-NETs

**Fig. 3:**
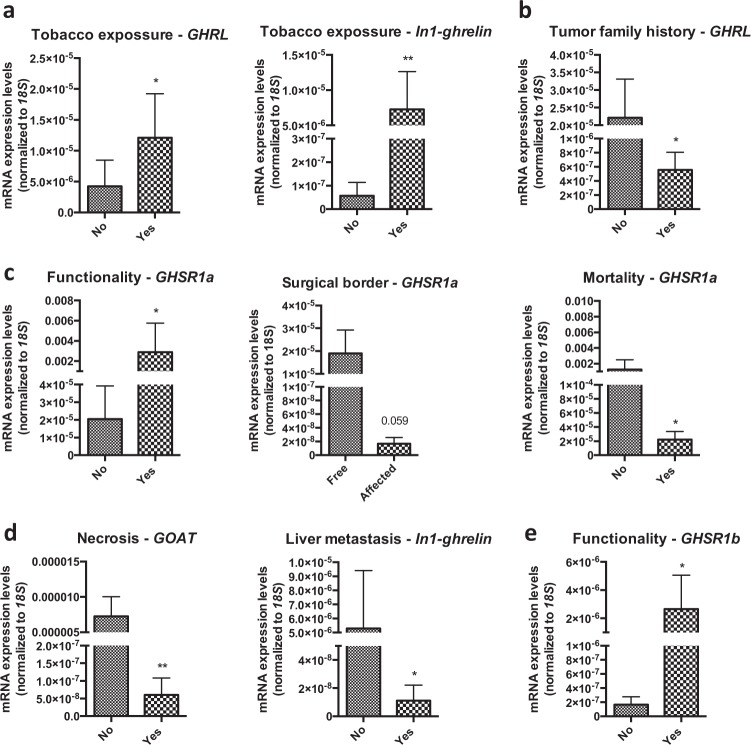
Correlations between epidemiological, clinical, histological, and molecular parameters in GEP-NETs. The correlations between epidemiological, clinical, histological, and molecular parameters within GEP-NET samples were assessed by *U*-Mann–Whitney tests. Asterisks indicate significant associations (*p < 0.05; **p < 0.01; ***p < 0.001)

Epidemiological data revealed that patients with tobacco exposure exhibited higher expression of ghrelin and In1-ghrelin (Fig. 3a). Moreover, patients with family history of tumor disease had a lower expression of ghrelin (Fig. 3b). Conversely, sex, personal history, previous neoplasm history, clinical symptoms, or other histological parameters (vascular/peritumoral invasion, lymph node metastasis) were not associated with the expression of any of the components of the ghrelin system.

Expression of some ghrelin system components was also associated to tumor characteristics, invasion capacity and prognosis in GEP-NETs. Specifically, functioning tumors presented higher levels of GHSR1a (Fig. 3c), while lower expression levels of this receptor were associated to the presence of affected surgical borders and mortality (Fig. 3c). Tumors with necrosis had lower GOAT mRNA levels and those with liver metastasis had decreased expression levels of In1-ghrelin (Fig. 3d). Interestingly, functionality was also associated with increased expression of GHSR1b (Fig. 3e). Finally, tumor diameter was directly correlated to GOAT expression (*R* = 0.33; *p* < 0.05). Remarkably, no further associations were found between expression levels of ghrelin system components and clinical/histological characteristics when considering separately PNETs and GI-NETs (data not shown).

### In vitro analysis of the role of GOAT in PNETs cell lines

**Fig. 4:**
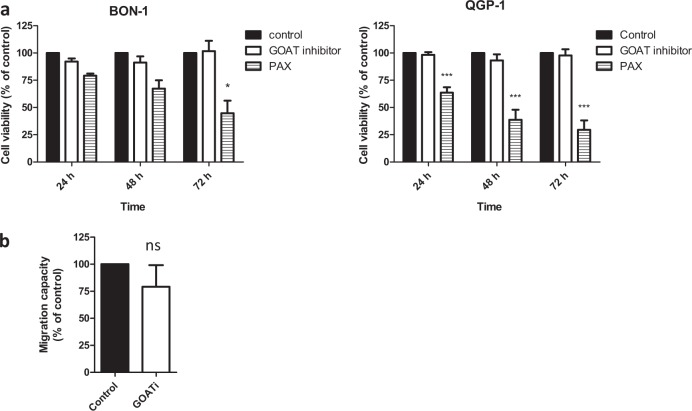
*I*n vitro analysis of the consequences of GOAT inhibitor (GOATi) treatment in NET cell lines. **a** Cell proliferation rate in BON-1 and QGP-1 cell lines after 24, 48, and 72 h of GOATi treatment determined by Alamar-blue assay. Paclitaxel (PAX) was used as inhibitory control in proliferation assays. **b** Cell migration rate in BON-1 after 24 h of treatment with GOAT inhibitor by wound-healing assay. Cell proliferation rate compared to control was assessed by multiple comparison tests while migration was assessed by *U*-Mann–Whitney test. Values represent the mean ± SEM of at least three individual experiments. Asterisks indicate significant differences (*p < 0.05; ***p < 0.001) compared with control (set at a 100%). Legend: ns means non-significant

We decided to further investigate the pathophysiological role of GOAT enzyme using the only available GOATi in PNETs cell lines. However, GOATi did not affect cell proliferation in BON-1 and QGP-1 cells (Fig. 4a) or the migration capacity of BON-1 cells (Fig. 4b).

### Discussion

This study aimed at evaluating systematically the expression of various components of the ghrelin system in an ample series of clinically well-characterized GEP-NETs, and to compare these expression levels with those in the corresponding adjacent non-tumor tissues and in normal control tissues. Previous studies have reported certain components of the ghrelin system in GEP-NETs^22,42–44;^ however, to our knowledge, this is the first study that comprehensively characterizes these components in tumor samples compared to their corresponding adjacent non-tumor regions, as well as with normal tissue samples. Moreover, we analyzed the demographic, epidemiological, and clinical characteristics as well as the disease progression and prognosis after 2–10 years of the patients with GEP-NETs. Overall, our results revealed that most of the components of the ghrelin system exhibit a distinctive expression in tumor and peritumoral tissues compared to normal tissue samples. Indeed, specific components of the ghrelin system, and especially GOAT, displayed remarkable alterations and clinical-histological correlations in tumor tissues, suggesting their potential value as novel biomarkers in GEP-NETs.

Also in our cohort, GEP-NETs exhibited a substantial molecular heterogeneity and variability^22,32,45^. Our results are consistent with previous reports showing that different components of the ghrelin system are present in tumor and non-adjacent tissues, and that, some of these components can be overexpressed in tumor samples compared to the surrounding tissue^22,42–44^. Differences among these studies may be related to the differences among patient cohorts.

Ghrelin system regulates key bodily functions, such as hormonal secretion and cell proliferation, in both normal and tumor cells^11–13,46,47^. In this context, our and other studies support the notion that the dysregulation of ghrelin system components observed in NETs could be pathologically relevant and may participate in tumor progression. The diverse localization and morphology of ghrelin-producing cells in the GI tract, and their implications on metabolic/endocrine functions, might suggest a role of this component in the regulation of GEP-NETs pathophysiology^12,13,42^, and could also explain the ample molecular heterogeneity found herein in the expression of ghrelin in different normal control tissues. Moreover, it could also be related to the overall overexpression of ghrelin in normal tissues compared to tumor samples. Although expression of canonical ghrelin has been described in various tumor types, its potential role in cancer is still controversial^48,49^. Ghrelin has been described in NETs using immunohistochemistry and qPCR^22,25,42,43^, in our cohort ghrelin was expressed in NET samples, albeit in substantially lower amounts than in normal tissues. In addition, in our cohort ghrelin expression levels were higher in PNETs than in GI-NETs, which is consistent with previous evidence^43^ but differed from other reports^44^. At variance with previous studies that did not find any clinical correlation between ghrelin expression and clinical features^22,42^, we observed here that ghrelin expression was higher in patients without tumor family history. Similarly, the in vitro effects of ghrelin on cell proliferation are also controversial^48,50–56^ and some studies have reported an association between ghrelin and poor survival in renal cell carcinoma patients^57,58^. Altogether, these data reinforce the notion that NETs are highly heterogeneous tumors, wherein the particular ghrelin expression profile and its clinical implications may depend on the type of tumor and the particular cohort of patients analyzed.

Expression of the canonical ghrelin receptor GHSR1a has been described in tumors including NETs^22,25,48^. Here, GHSR1a expression was highly variable in normal control samples, but tended to be overexpressed in tumor samples compared to adjacent non-tumor tissue, which is consistent with our previous study in a different cohort^22^. The relation between GHSR1a, functionality and mortality invites to explore further the potential relationship of this receptor with tumorigenesis, and its putative value as a molecular prognostic marker in NETs.

The pathophysiological implications of the ghrelin system have been recently expanded with the discovery of new molecular components^11,16,17,29^, which have been found to be overexpressed in several tumors^16,22,27^ and associated to relevant clinical parameters^22^. Herein we found comparable tendencies in the expression of these variants; however, these differences did not reach statistical significance. Nevertheless, in the present cohort, >40% of tumor samples presented detectable levels of In1-ghrelin and GHSR1b, while in the previous study, >80% of the tumor samples exhibited detectable levels^22^. These differences could likely reduce the statistical power of the comparisons and correlations, and, again, would illustrate the elevated heterogeneity of NETs.

The most novel and relevant finding of this study is the marked overexpression of GOAT in NET samples. Whereas the expression of this enzyme was almost absent in control tissues, it was present in adjacent non-tumor tissue and notably overexpressed in tumor tissues. These, together with previous results showing a similar, remarkable overexpression of GOAT in breast and pituitary tumors^11,21^ provide suggestive evidence for a striking dysregulation of this enzyme in endocrine-related tumors. The expression levels GOAT does not always correlate with those of ghrelin, whereas they do parallel more consistently the expression levels of In1-ghrelin, suggesting the existence of additional targets for GOAT enzyme^11^. In NETs, GOAT levels have been correlated with those of In1-ghrelin, and associated with worse outcome^22^, these findings were not reproducible in our cohort, which may be explained by the tumor heterogeneity and the limited number of tumor samples. Despite this, in the present study, GOAT expression is associated to larger tumors, especially in GI-NETs, reinforcing the notion of a possible association between the dysregulation of this enzyme and the pathophysiology of NETs. This is the first study that demonstrates an intense overexpression of GOAT enzyme by IHC in GEP-NETs tissues compared to non-tumor adjacent tissues; however, its functional implications should be precisely defined. Here, a GOAT inhibitor administered on two NET cell lines, BON-1 and QGP-1, did not show relevant changes in cell proliferation or migration in vitro. Thus, future studies should explore this further, using novel inhibitors or other inhibiting/silencing approaches.

Notwithstanding this, our current and previous^22^ studies provide compelling evidence that certain components of the ghrelin system, and specially GOAT enzyme, are clearly overexpressed in NETs, suggesting their potential value as diagnostic and/or prognostic biomarkers for this pathology. In support of the present finding in NETs, GOAT has been also recently reported as non-invasive plasma biomarker in prostate cancer^18^. Additionally, the association between GHSR1a and GHSR1b with the functionally of these tumors and the mortality of these patients further supports this notion and emphasizes the importance of exploring the modulation of this receptor for improving patient outcome. Therefore, although it is difficult to predict the specific clinical impact of these findings, taken together, all these results invite to analyze in more detail the putative utility of GOAT overexpression as a diagnostic biomarker in NETs.

In summary, we present the first systematic characterization of the components of the ghrelin system, including splicing variants, in GEP-NETs tissues in comparison with their adjacent non-tumor regions, and also with normal tissue samples. Our results demonstrate that key components of this system are markedly dysregulated in GEP-NETs and associated to key clinical parameters, suggesting the interest of further studying these molecular targets, especially GOAT, as putative diagnosis and/or prognostic markers in GEP-NETs.

## Study highlights

### What is current knowledge

– Some components of ghrelin system could be altered in neuroendocrine tumors

### What Is New Here

– Key components of ghrelin system are markedly dysregulated in GEP-NETs and associated to key clinical parameters.
– Changes in the expression of ghrelin system components are associated with the development and/or progression of GEP-NETs.
– These molecular targets, especially GOAT, may represent putative diagnosis and/or prognostic markers in GEP-NETs.

## Electronic supplementary material

Supplemental Figures

Supplemental Figure legends
