# Supplementary figures and images for: Ghrelin-O-Acyltransferase (GOAT) Enzyme as a Novel Potential Biomarker in Gastroenteropancreatic Neuroendocrine Tumors
Source: Clin Transl Gastroenterol. 2018 Oct 8;9(10):196. doi: 10.1038/s41424-018-0058-8 (PMC6175927; doi:10.1038/s41424-018-0058-8)

# Supplemental Figure 1

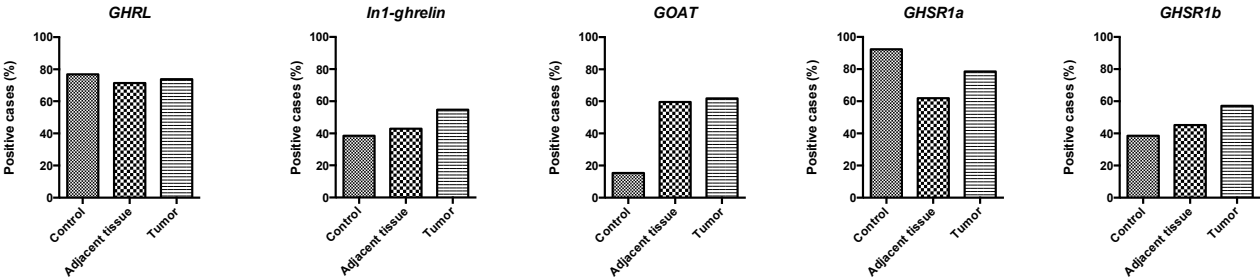

# Supplemental Figure 2

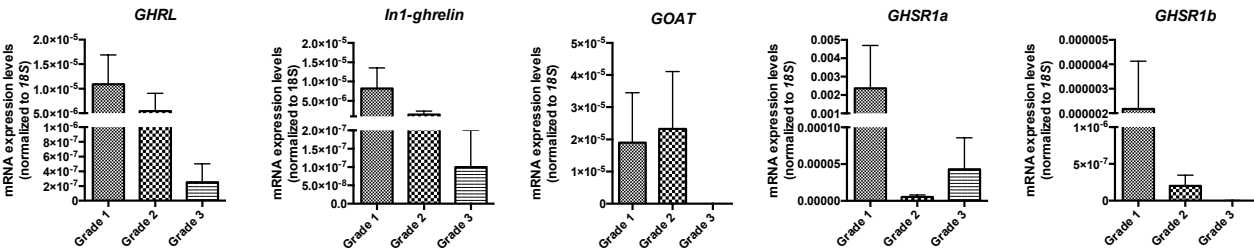

Supplemental Figure 3

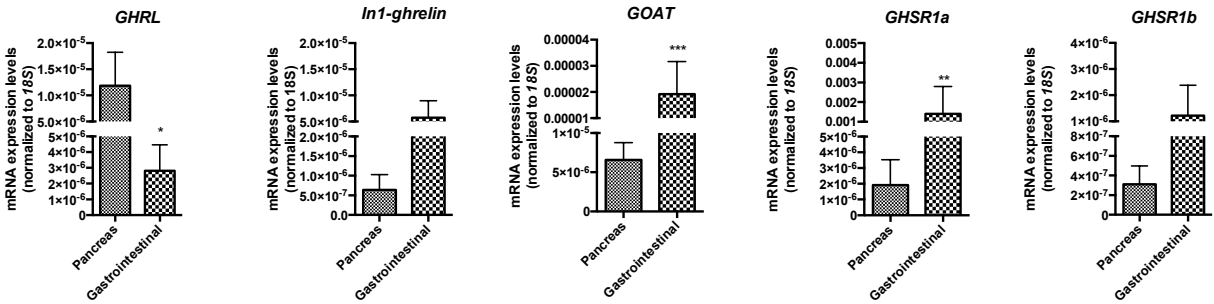

Supplement: Supplementary file 1 — Supplemental Figures [file 41424_2018_58_MOESM1_ESM.pdf]
