# Supplementary material for: Ghrelin-O-Acyltransferase (GOAT) Enzyme as a Novel Potential Biomarker in Gastroenteropancreatic Neuroendocrine Tumors
Source: Clin Transl Gastroenterol. 2018 Oct 8;9(10):196. doi: 10.1038/s41424-018-0058-8 (PMC6175927; doi:10.1038/s41424-018-0058-8)
Supplement: Supplementary file 2 — Supplemental Figure legends [file 41424_2018_58_MOESM2_ESM.docx]

**Supplemental Figure 1: Presence of ghrelin system components in normal GEP, adjacent non-tumor tissue and GEP-NETs.** The graphs indicate the percentage of samples (normal GEP control, adjacent non-tumor tissue and tumoral tissue) positive for the expression of each of the ghrelin system components.

**Supplemental Figure 2: Expression of ghrelin system components in GEP-NETs of different grade.** The absolute mRNA expression of the different components of the ghrelin system was analyzed in grade 1, 2 and 3 GEP-NETs samples (values are adjusted by 18S expression). Data represent the mean ± SEM.

**Supplemental Figure 3: Expression of ghrelin system components in pancreas and gastrointestinal NETs.** The absolute mRNA expression of the different components of the ghrelin system was analyzed in pancreas and gastrointestinal NETs samples. Data represent the mean±SEM. Asterisks (**,p<0.01; ***, p<0.001) indicate significant changes by non-paired analysis.
